# Relationship between hemoglobin and grip strength in older adults: the ActiFE study

**DOI:** 10.1007/s40520-024-02698-7

**Published:** 2024-03-07

**Authors:** Theresa Hammer, Ulrike Braisch, Dietrich Rothenbacher, Michael Denkinger, Dhayana Dallmeier

**Affiliations:** 1 Research Unit on Ageing at Agaplesion Bethesda Clinic Ulm, Ulm, Germany; 2 Institute for Geriatric Research, Ulm University, Ulm, Germany; 3 Institute of Epidemiology and Medical Biometry, Ulm University, Ulm, Germany; 4 Medical Faculty, Ulm University, Ulm, Germany; 5 Department of Epidemiology, Boston University School of Public Health, Boston, USA

**Keywords:** Grip strength, Hemoglobin, Iron status, Ferritin, Older adults

## Abstract

**Introduction:**

Although anemia is associated with low muscle strength, hemoglobin has been rarely studied considering ferritin.

**Aim:**

To analyze the association between hemoglobin and grip strength in community-dwelling older adults.

**Methods:**

We used data from a German cohort of adults ≥ 65 years, excluding those with CRP > 10 mg/L or taking iron supplements. Grip strength (kg) was measured using a Jamar dynamometer. Analysis was performed using multiple linear regression, adjusted for established confounders. Due to interaction, age-stratified (< 80, 80 +), further sex-stratified analysis in those < 80 years old and ferritin-stratified in men < 80 years were performed.

**Results:**

In total, 1294 participants were included in this analysis (mean age 75.5 years, 549 (42.3%) women, 910 (70.3%) < 80 years). On average, hemoglobin and grip strength were 14.9 g/dL and 41.3 kg for men, 13.9 g/dL and 25.1 kg for women. Hemoglobin was significantly positively associated with grip strength only among women < 80 years (β 0.923 [95% CI 0.196, 1.650])*.* For men < 80 years, the association was significant when ferritin was ≥ 300 µg/L (β 2.028 [95% CI 0.910, 3.146]). No association was detected among those participants 80 + .

**Discussion and conclusions:**

Our data show an association between hemoglobin and grip strength only in women < 80 years old. For men < 80 years, the association was only significant with ferritin levels ≥ 300 µg/L. Considering the decreasing levels of hemoglobin and grip strength and the high prevalence of iron deficiency in older adults further analyses investigating this relationship with more iron specific parameters such as transferrin saturation are warranted.

**Supplementary Information:**

The online version contains supplementary material available at 10.1007/s40520-024-02698-7.

## Introduction

Grip strength is a well-established marker for physical condition and a surrogate for the overall muscle strength. The European Working Group on Sarcopenia in Older People 2 recommends the measurement of hand grip strength for identifying low muscle strength [1]. Grip strength can also be used as a surrogate marker in the diagnosis of functional frailty and as a prognostic marker for ageing. Diminished grip strength has been associated with an increased risk for mortality, disability, various complications and adverse health outcomes, such as longer hospital stays [2–7]. At the same time, declining levels of hemoglobin have been reported over time even in older adults without anemia [8]. Additionally, grip strength as well as hemoglobin are easy and cheap to measure in the clinical and ambulatory settings.

In this context, the prevalence of iron deficiency, one of the main pathophysiological mechanism behind low hemoglobin levels, has been reported as high as 26.8% according to findings from the DO-HEALTH study including healthy community-dwelling participants aged ≥ 70 years in five European Countries (Austria, Switzerland, Germany, Portugal, France), with a higher prevalence in women as compared to men [9]. In hospitalized patients, the prevalence of iron deficiency, representing the end-stage of iron deficiency, has been reported to be as high as 58% [10–12].

The human body contains around 30–40 mg iron per kilogram bodyweight [13], with 10–15% of the total iron located in the skeletal muscle as part of myoglobin [14], and another significant part bound to hemoglobin. Iron plays an important part for getting oxygen to terminal oxidases, it is part of the active site in cytochrome molecules and is also present in the respiratory chain [13]. The association of anemia per se or hemoglobin with various functionality parameters has already been often investigated. In this context, iron deficiency is a common condition affecting more than two billion people worldwide, with a well-established association with anemia [15]. Iron replacement therapy has shown significant clinical improvement in patients with heart failure and iron deficiency with ferritin levels < 300 µg/L and transferrin saturation < 20% [16–18]. These observations lead to the hypothesis about a possible interplay between hemoglobin and ferritin in the association between hemoglobin and other functional parameters such as grip strength in older adults. Therefore, the aim of this study was to analyze the association between hemoglobin and grip strength considering ferritin, an indirect marker for the storage of iron in the body, in a cohort of community-dwelling older people.

## Methods

### Study population

The ActiFE study (Activity and Function in the Elderly in Ulm) is a population-based cohort study in Ulm and surrounding areas located in Southern Germany. All community-dwelling older adults, aged ≥ 65 years, able to walk through their own room, randomly selected from the local residents` registration office between March 2009 and April 2010 could be considered for participation. Those randomly selected citizens received a letter of invitation for participation in the study via mail. Exclusion criteria were difficulties in understanding German, severe cognitive deficits, which would not allow the person to accomplish the assessments or being in residential care. The study population consisted originally of 1506 participants (overall response 20%). Details of the study are published elsewhere [19]. For this analysis, those with missing information for hemoglobin (n = 22), ferritin (n = 8), grip strength (n = 34) or for any of the possible confounders identified in the literature and used in this analysis were excluded (education (n = 15), living alone (n = 18), smoking (n = 1), alcohol consumption (n = 26), C-reactive Protein (n = 6), Cystatin-C GFR (n = 6), body-mass-index (n = 9). Because ferritin can be also considered as an unspecific inflammation marker, those participants with high-sensitive C-reactive protein (hs-CRP) ≥ 10 mg/L at baseline, representing an underlying inflammatory process, were also excluded (n = 73). In addition, participants taking iron supplementation at baseline (n = 3) were also excluded. In total, 1294 participants remained for this analysis.

### Measurements

Baseline assessments were done by trained research assistants using standardized questionnaires and instruments. The following socio-demographic covariates were attained via self-report: age, sex, education (> 10 or ≤ 10 years) and the status of living alone (yes/no). Information on established confounders were included as follows: alcohol consumption categorized in four groups (daily alcohol intake, intake several times per week, several times per month or less than once a month), smoking classified in three groups (current smokers, former smokers and never smokers), polypharmacy defined as the simultaneous intake of ≥ 5 drugs (yes/no). Additionally, body-mass-index was calculated from measured height (in meters) and weight (in kilograms).

Venous blood was drawn, centrifuged, aliquoted and frozen at − 80 °C under standardized conditions. Analyzed blood parameters included hemoglobin (g/dL), ferritin (µg/L), cystatin C (mg/L) and hs-CRP (mg/L). Hemoglobin was measured via photometry, with a ref. range of 4.4–11.3 Giga/L. Ferritin was estimated using a TURBID method. Cystatin C and hs-CRP were measured via Nephelometrie BNII, Fa. Siemens (cystatin C measuring range 0.0031–0.0995 mg/L, CV % 2.21, hs-CRP measuring range 0.17–1100, Intra-Assy Precision 3.57%). Glomerular filtration rate (mL/min/1.73 m^2^) was calculated using the CKD-EPI Formula for cystatin C [20].

Grip strength was measured in kilograms using a JAMAR Dynamometer (Sammons, Preston, Bolingbrook, Illinois). The strength of each hand was measured twice, the mean for each side was calculated and the higher mean value was used in this analysis. This approach has been chosen, in order to adjust for the possible bias of obtaining higher values observed by the performance of serial measurements [21].

### Statistical analysis

For descriptive analysis, metric variables are reported as mean ± standard deviation (SD) or median with interquartile range (Q1, Q3) in accordance to their distribution. Categorical variables are expressed with absolute numbers and their respective percentages. For the identification of relevant confounders univariable regression was used to examine the crude associations between hemoglobin level and grip strength with identified covariables possible relevant for this relationship as well as for additional covariables identified in the literature. Correlation between covariates was examined using Spearman coefficients. Ferritin levels and hs-CRP were log-transformed for having a not normal distribution. Outliers were identified using Cook’s distance and optic recognition and excluded from the analysis (maximally excluded per analysis: n = 2).

We performed a multiple linear regression adjusted initially for age and sex (Model 1), with further adjustment for the following identified confounders living alone, smoking, alcohol consumption, level of education, polypharmacy, BMI, eGFR, log (ferritin) and log (hs-CRP) (Model 2). We examined the presence of effect modification by age and sex (interaction terms Hb*age and Hb*sex, respectively). A p-value of < 0.2 was considered as an indication for possible effect modification, followed by a further evaluation using stratified analysis [22]. We detected an interaction with age (p = 0.002); therefore, analyses were performed stratified as follows: < 80 versus ≥ 80 years old, in order to differentiate between the old and the very old adults. A further interaction by sex was identified by those < 80 years old (p = 0.090), leading to a sex-stratified analysis in this age-group. An interaction with ferritin was also identified only among men < 80 years old (p = 0.015), therefore the analysis was stratified according to the ferritin levels as follows: < 100 µg/L, ≥ 100 up to < 300 µg/L, ≥ 300 µg/L. Those cut-off values were chosen in analogy to the cut-off values applied in the diagnosis of iron deficiency in heart failure [23]. Additionally, we performed a sensitivity analysis excluding participants with hs-CRP ≥ 5 mg/dL (n = 163). All analyses were performed using SPSS Versions 27 und 29. The graphs were generated using R software (Version 4.2.2).

## Results

Table 1 shows the baseline characteristics of our study population. On average, they were 75.5 (± 6.5) years old, 42.3% were female. The mean grip strength in the overall population was 38.73 (± 9.35) kg for men and 23.99 (± 7.14) kg for women. The average hemoglobin level was 14.32 (± 1.18) g/dL, with 14.70 (± 1.18) g/dL in men, and 13.81 (± 0.97) g/dL in women. Overall, only 73 participants were anemic (5.6%). Those < 80 years old were more likely to be female, to be current smokers and to not live alone than those 80 + . They were more likely to have higher grip strength, higher levels of hemoglobin and a lower rate of anemia. Furthermore, they had higher ferritin levels, a higher glomerular filtration rate, a lower level of hs-CRP and they were less likely to be on polypharmacy.

**Table 1:** Baseline characteristics of the study population

	Overall (n = 1294)	< 80 years (n = 910)	80 + years (n = 384)
Age (years), mean (SD)			
Overall	75.5 (6.5)	71.9 (3.8)	83.9 (2.6)
Men	75.9 (6.4)	72.0 (3.7)	83.5 (2.5)
Women	74.8 (6.5)	71.8 (3.8)	84.6 (2.8)
Female, n (%)	548 (42.3)	418 (45.9)	130 (33.9)
Smoking, n (%)			
Never	643 (49.7)	463 (50.9)	180 (46.9)
Former	559 (43.2)	368 (40.4)	191 (49.7)
Current	92 (7.1)	79 (8.7)	13 (3.4)
Alcohol consumption, n (%)			
Daily	406 (31.4)	275 (30.2)	131 (34.1)
Several times per week	346 (26.7)	255 (28.0)	91 (23.7)
Several times per month	318 (24.6)	230 (25.3)	88 (22.9)
Less than once per month	224 (17.3)	150 (16.5)	74 (19.3)
Education ≤ 10 years, n (%)	1024 (79.1)	718 (78.9)	306 (79.7)
Living alone, n (%)	308 (23.8)	176 (19.3)	132 (34.4)
Handgrip strength (kg), mean (SD)			
Men	38.73 (9.35)	41.33 (9.09)	33.70 (7.21)
Women	23.99 (7.14)	25.06 (7.13)	20.53 (5.98)
Hemoglobin (g/dL), mean (SD)			
Overall	14.32 (1.18)	14.44 (1.13)	14.04 (1.25)
Men	14.70 (1.18)	14.90 (1.11)	14.31 (1.22)
Women	13.81 (0.97)	13.91 (0.89)	13.49 (1.14)
Anemia^a^, n (%)			
Overall	73 (5.6)	30 (3.3)	43 (11.2)
Men	56 (7.5)	24 (4.9)	32 (12.6)
Women	17 (3.1)	6 (1.4)	11 (8.5)
Ferritin (µg/L), median (Q1, Q3)			
Overall	154.00 (88.00, 259.25)	157.00 (94.00, 268.00)	141.00 (76.25, 246.00)
Men	185.00 (110.75, 307.00)	194.50 (117.25, 324.25)	167.00 (93.00, 274.25)
Women	119.00 (70.00, 190.00)	126.00 (80.00, 200.50)	96.00 (49.75, 175.75)
Ferritin, n (%)			
< 100 (µg/L)	388 (30.0)	252 (27.7)	136 (35.4)
≥ 100—< 300 (µg/L)	657 (50.8)	472 (51.9)	185 (48.2)
≥ 300 (µg/L)	249 (19.2)	186 (20.4)	63 (16.4)
BMI (kg/m^2^), mean (SD)	27.47 (4.00)	27.56 (4.03)	27.28 (3.92)
Cystatin eGFR (mL/min), mean (SD)	82.17 (19.74)	87.80 (17.33)	68.82 (18.67)
hs-CRP (mg/L), median (Q1, Q3)	1.63 (0.84, 3.22)	1.57 (0.83, 3.01)	1.89 (0.86, 3.97)
Polypharmacy, n (%)	378 (29.2)	201 (22.1)	177 (46.1)
Falls in the last 12 months, n (%)	449 (34.7)	298 (32.7)	151 (39.4)
Falls in the last 3 months, n (%)	219 (16.9)	142 (15.6)	77 (20.1)
Frailty Index^b^ ≥ 0.2, n (%)	225 (20.4)	103 (13.1)	122 (38.7)

^a^Anemia according to WHO-definition: < 12 g/dL (women), < 13 g/dL (men)

^b^Frailty index [44] is provided for characterization of the sample, and was available overall for 1104 (85.3%) participants (n = 780 < 80 years old, n = 315 80 +)

### Hemoglobin and hand grip strength in those < 80 years

**Fig. 1:**
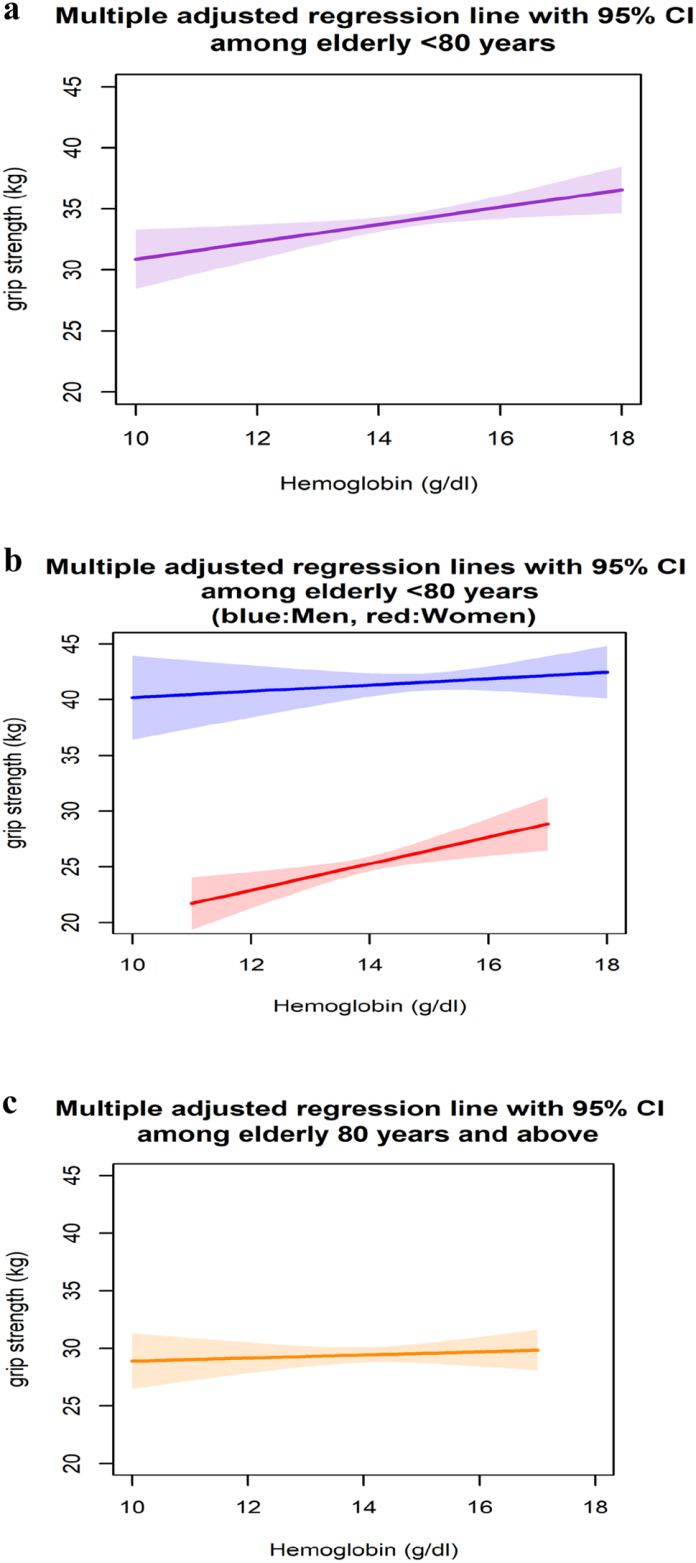
Multiple adjusted regression lines with 95% CI for the association between hemoglobin and grip strength adjusted for age, sex, log(ferritin), log(hs-CRP), smoking, alcohol, eGFR, BMI, polypharmacy, education, living alone among those a) < 80 years, b) < 80 years stratified by sex (blue/above = male, red/below = female), c) 80 +

In women < 80 years old (n = 416), there was a statistically significant positive association between hemoglobin and grip strength, which was slightly lessened after adjusting for further covariates so that an increment of one gram hemoglobin was statistically significantly associated with an increment of 0.923 kg in grip strength [95% CI 0.196, 1.650] (Table 2, Fig. 1b). We did not detect an interaction with ferritin among women.

**Table 2:** Linear regression models for the association between hemoglobin and grip strength among participants < 80 years old stratified by sex

	n	Model 1^a^ β [95% CI]	Model 2^b^ β [95% CI]
Women	416	**1.058 [0.358, 1.759]**	**0.923 [0.196, 1.650]**
Men	491	0.467 [− 0.228, 1.163]	0.130 [− 0.554, 0.814]

Bold highlights the statisically significant associations

^a^ adjusted for age

^b^ adjusted for age, log(ferritin), log(hs-CRP), smoking, alcohol, eGFR, BMI, polypharmacy, education, living alone

In men < 80 years old (n = 491), an interaction between hemoglobin and ferritin levels was detected. A statistically significant positive association between hemoglobin and hand grip could be detected only among those with ferritin ≥ 300 µg/L, where an increment of one gram hemoglobin was statistically significantly associated with an increment of 2.028 kg in grip strength [95% CI 0.910, 3.146] (Model 2) (Table 3).

**Table 3:** Linear regression models for the association between hemoglobin and grip strength among male participants < 80 years old stratified by ferritin tertiles

	n	Model 1^a^ β [95% CI]	Model 2^b^ β [95% CI]
Ferritin < 100 µg/L	99	− 0.797 [− 2.249, 0.654]	− 1.079 [− 2.249, 0.654]
Ferritin ≥ 100 & < 300 µg/L	249	−0.352 [− 1.560, 0.856]	− 0.330 [− 1.551, 0.891]
Ferritin ≥ 300 µg/L	144	**2.181 [1.180, 3.182]**	**2.028 [0.910, 3.146]**

Bold highlights the statisically significant associations

^a^adjusted for age

^b^adjusted for age, log(ferritin), log(hs-CRP), smoking, alcohol, GFR, BMI, polypharmacy, education, living alone

### Hemoglobin and hand grip strength in those 80 + 

In those 80 + (n = 382), neither an interaction with ferritin nor a statistically significant association between hemoglobin and grip strength was observed (Table 4, Fig. 1c).

**Table 4:** Linear regression models for the association between hemoglobin and grip strength among participants 80 + years old (β estimates, [95% CI])

	n	Model 1^a^	Model 2^b^
Overall	382	0.241 [− 0.310, 0.792]	0.173 [− 0.398, 0.745]

^a^adjusted for age and sex

^b^adjusted for age, sex, log(ferritin), log(hs-CRP), smoking, alcohol, eGFR, BMI, polypharmacy, education, living alone

### Sensitivity analysis

When restricting our analysis to those with a hs-CRP < 5 mg/dL (n = 1131), the association in women < 80 years not only remained significant but also showed a stronger association (model 2: β = 1.293 [95% CI 0.460, 2.126]) (Supplementary Table 1). For men < 80 years, the interaction between hemoglobin and ferritin remained significant, and only those men with ferritin ≥ 300 µg/L showed a significant association meaning an increment of one gram of hemoglobin was significantly associated with an increment of 2.559 kg in grip strength (β = 2.559 [95% CI 1.443, 3.674]) (Supplementary Table 2). In those 80 + (n = 316), no statistically significant association between hemoglobin and grip strength was observed (Supplementary Table 3).

## Discussion

Our data based on a study population of 1294 community-dwelling adults aged 65 years and older showed a statistically significant association between hemoglobin and grip strength only among those < 80 years old. Furthermore, we detected an interaction with ferritin, an indirect marker for storage of iron, only in men, so that one gram increment in hemoglobin was associated with an improvement in grip strength only among men with ferritin levels ≥ 300 µg/L. Contrary to our expectations, we did not find a significant association between hemoglobin and grip strength in those ≥ 80 years, nor an interaction with ferritin which may partly be explained by the relatively small sample size in this age group. Further analyses in larger cohorts are warranted.

We observed overall a low prevalence of anemia in our study population when compared to the WHO estimations for this age group. However, this low prevalence and the observed mean grip strength values are comparable to other European cohorts. Results from the nationwide German Health Interview and Examination Survey for Adults (DEGS1), a cross-sectional study conducted among participants aged 65 to 79 years (n = 1774), show similar sex-specific mean grip strength values [24]. Our mean grip strength values are also in line with those of other cohorts such as Health, Aging and Body Composition Study Cohort and NHANES [2, 25].

So far, few studies have investigated the association between anemia and functionality parameters. Thus far, the role of hemoglobin, even across the range of normal values, remains unclear as they provided partly divergent results. In a Taiwanese study, hemoglobin was associated with low grip strength and slowness, but not with sarcopenia [34]. However, there was no adjustment for inflammation and iron indices were not reported. In a population of non-anemic participants, a decline in hemoglobin levels or incident anemia were associated with mortality, specially strong among those with low hemoglobin levels Although there was a significant association between hemoglobin and grip strength for men and women at baseline, the association between incident anemia or decline in hemoglobin levels and grip strength was not significant [8].

Until now, only few studies have performed sex-stratified analyses in this context [35]. In our study, we did find an effect modification by sex (Fig. 1). Associations were significant for women < 80 years old, and for men < 80 years old with a Ferritin level ≥ 300 µg/L. So far, available literature is controversial. One study supports our findings in a population with a high prevalence of anemia (40%), showing a significant association with grip strength in women, but not in men, even after adjustment for chronic inflammation [36]. Unfortunately, iron status was not considered. Another study did demonstrate only in men a significant association between hemoglobin and grip strength, both in cross-sectional and longitudinal analysis [37].

With respect to further iron indices, a recent study investigated the association with grip strength in 477 community-dwelling adults aged ≥ 65 years, characterized by a high prevalence of low muscle strength. They found that ferritin as well as transferrin saturation were significant predictor variables for grip strength even after adjustment for hemoglobin and hs-CRP. Unfortunately, the presence of effect modification by sex was not evaluated [38].

Iron deficiency is a common phenomenon, even in high-income countries with low rates of anemia [9]. Overall, the relationship of iron deficiency measures with functional parameters shows conflicting results. An Australian study reported that maintaining or increasing the total iron intake was associated with reduced risk of developing frailty [39]. These findings were only true for the physical frailty definition according to Fried, not for the deficit accumulation index by Rockwood. In a small cohort of healthy 149 community-dwelling participants aged 55 years and more in Portugal with CRP-levels < 10 mg/L, a significant association between iron deficiency and functional tiredness and erythropoiesis was found. This was, however, not the case for parameters for iron storage and transport. [40]. A study from South Korea found a significant association between elevated ferritin and the prevalence of sarcopenia in older women, but not in men [41].

With respect to more vulnerable, hospitalized older patients, a recent study in 224 hospitalized patients aged 65–95 from Germany investigated the association of iron deficiency, fatigue, and muscle strength [11]. They identified iron deficiency as a risk factor for fatigue and poor functional recovery and suggested that supplementing iron might allow a better improvement in functionality. This approach would be in analogy to the current recommendations for iron supplementation by patients with heart failure and iron deficiency with ferritin levels < 300 µg/L and transferrin saturation < 20% [23]. Data from FAIR-HF showed for iron-deficient patients with chronic heart failure, both anemic and non-anemic, an improvement in the NYHA rating and the 6 min walk test without significant differences in safety endpoints as compared to placebo [16, 17]. In addition, AFFIRM-HF, a multi-center, randomized trial with iron-deficient patients with acute heart failure demonstrated an improvement in hospitalization rate, and days lost to hospitalizations in those treated with iron compared to placebo [18]. A further validation of our results would support among others the need to assess routinely ferritin levels as a surrogate for iron storage, even in those without anemia, to identify those with iron deficiency. Considering the higher prevalence of i) blood loss, also in the settings of the increasing surgical treatment of hip fractures, ii) poor iron intake, iii) malabsorption of iron, as well as of iv) anemia of chronic disease in older adults [42], the further evaluation of the interplay between iron deficiency, related anemia and functionality in older adults should be prospectively investigated independent of acute or chronic heart failure. Clinical trials should in this context evaluate the hypothesis of a possible better and faster improvement of functional parameters after iron supplementation, regardless of the presence of anemia, in those subjects with iron deficiency in comparison to those without iron supplementation. Should the hypothesis of a better and faster recovery after iron supplementation among those with iron deficiency, even in the absence of anemia, being confirmed, this could open new windows for therapeutic options to support the rehabilitation process and increase functional recovery of older adults.

### Strengths and limitations

This study has some limitations. Firstly, only relatively mobile community-dwelling participants were included in this study. Therefore, these participants were most likely healthier and fitter than the general population in the same age range. This might explain the low prevalence of anemia in our study population. However, the prevalence of age relevant conditions such as frailty and falls in the previous year as well as the distribution of other biomarkers such as NT-proBNP [43], are consistent with the one reported in other population-based studies [44, 45]. Secondly, due to the study design of the ActiFE study, there was an oversampling of younger participants. This might affect our power to detect any effect modification by sex and/or ferritin in those 80 + . However, the ActiFE cohort represents a well-characterized middle size study population with respect to clinical parameters as well as blood biomarkers, allowing the performance of stratified and secondary analyses. Because ferritin is an acute phase protein, we excluded participants with a hs-CRP level ≥ 10 mg/L to prevent underlying inflammatory conditions potentially distorting our analyses. Unfortunately, we did not have data on iron levels at baseline. Therefore, we were not able to use transferrin saturation as a marker for iron’s storage. However, and considering the fact that only very few of the excluded subjects were taken iron supplementation one can assume the absence of severe iron deficiency states in our population, allowing the focus on the association between hemoglobin and hand grip strength.

## Conclusion

Our data showed a statistically significant positive association between hemoglobin and grip strength only among women < 80 years old, independently of ferritin levels. For men the association was only statistically significant among those < 80 years with ferritin levels ≥ 300 µg/L. Considering the declining levels of hemoglobin in older adults, the observed decreased hand grip strength with a well-established association with incident disability, as well as considering the increasing prevalence of malabsorption and iron deficiency in this population further analyses and clinical trials investigating the interplay between iron storage and functional parameters such as grip strength even in the absence of anemia are warranted.

### Supplementary Information

Below is the link to the electronic supplementary material.

Supplementary file1 (PDF 124 KB)

## Data Availability

The datasets analyzed during the current study are not publicly available due to the conditions provided in the informed consent at the time of recruitment but are available from the corresponding author on reasonable request.
