# Supplementary material for: Relationship between hemoglobin and grip strength in older adults: the ActiFE study
Source: Aging Clin Exp Res. 2024 Mar 7;36(1):59. doi: 10.1007/s40520-024-02698-7 (PMC10920471; doi:10.1007/s40520-024-02698-7)
Supplement: Supplementary file 1 — Supplementary file1 (PDF 124 KB) [file 40520_2024_2698_MOESM1_ESM.pdf]

Supplementary Data: Relationship between hemoglobin and grip strength in older adults – the ActiFE Study

Theresa Hammer[1,2], Ulrike Braisch[1,3], Dietrich Rothenbacher[3] (ORCID ID 0000-0002-3563-2791), Michael Denkinger[1,2] (ORCID ID 0000-0002-8097-060X), Dhayana Dallmeier[1,4] (ORCID ID 0000-0003-3665-7023).

1 Research Unit on Ageing at Agaplesion Bethesda Clinic Ulm, Ulm, Germany

2 Institute for Geriatric Research, Ulm University, Ulm, Germany

3 Institute of Epidemiology and Medical Biometry, Ulm University, Ulm, Germany

4 Department of Epidemiology, Boston University School of Public Health, Boston, USA

Supplementary Material

Suppl. Table 1: Linear regression models for the association between grip strength and hemoglobin among participants <80 years old and hs-CRP <5 mg/L, stratified by sex.

	n	Model 1*<br>ß [95 CI]	Model 2**<br>ß [95 CI]
Women	374	1.461 [0.644, 2.266]	1.293 [0.460, 2.126]
Men	433	0.233 [-0.553, 1.019]	-0.003 [-0.810, 0.803]

* adjusted for age.

** adjusted for age, log(ferritin), log(hs-CRP), smoking, alcohol, eGFR, BMI, polypharmacy, education, living alone.

Suppl. Table 2: Linear regression models for the association between grip strength and hemoglobin among male participants <80 years old and hs-CRP <5 mg/L, stratified by ferritin-tertiles.

	n	Model 1*<br>ß [95 CI]	Model 2**<br>ß [95 CI]
Ferritin <100 µg/L	94	-0.916 [-2.528, 0.695]	-0.973 [-2.821, 0.876]
Ferritin ≥100 & <300 µg/L	225	-0.852 [-2.117, 0.413]	-0.855 [-2.147, 0.438]
Ferritin ≥300 µg/L	124	2.559 [1.443, 3.674]	2.728 [1.535, 3.921]

* adjusted for age.

** adjusted for age, log(ferritin), log(hs-CRP), smoking, alcohol, eGFR, BMI, polypharmacy, education, living alone.

Suppl. Table 3 Linear regression models for the association between grip strength and hemoglobin among participants 80+ years old and hs-CRP <5 mg/L.

	n	Model 1*<br>ß [95 CI]	Model 2**<br>ß [95 CI]
Overall	316	0.176 [-0.424, 0.758]	0.097 [-0.524, 0.718]

* adjusted for age and sex.

** adjusted for age, sex, log(ferritin), log(hs-CRP), smoking, alcohol, eGFR, BMI, polypharmacy, education, living alone.
